# Postural Sway in Lower Extremity Amputees and Older Adults May Suggest Increased Fall Risk in Amputees

**DOI:** 10.33137/cpoj.v3i2.33804

**Published:** 2020-09-20

**Authors:** H. Bateni

**Affiliations:** Physical Therapy Program, School of Allied Health and Communicative Disorders, Northern Illinois University, DeKalb, Illinois, USA.

**Keywords:** Amputation, Postural balance, Amputee, Prosthesis, Lower limb amputation, Postural sway

## Abstract

**BACKGROUND:**

Falls can be detrimental to overall health and quality of life for lower extremity amputees. Most previous studies of postural steadiness focus on quantification of time series variables extracted from postural sway signals. While it has been suggested that frequency domain variables can provide more valuable information, few current studies have evaluated postural sway in amputees using frequency domain variables.

**METHODOLOGY:**

Participants were assigned to 3 groups: lower extremity amputation (n=6), healthy young adults (n=10), and healthy older adults (n=10). Standing barefoot on a force platform, each individual completed 3 trials of each of 3 standing conditions: eyes open, eyes closed, and standing on a foam balance pad. Time and frequency domain variables of postural sway were computed and analyzed.

**RESULTS:**

Comparison of older adults, younger adults, and amputees on the three conditions of standing eyes open, eyes closed, and on foam revealed significant differences between groups. Mean mediolateral (ML) sway distance from the center of pressure (COP), total excursions and sway velocity was significantly higher for amputees and older adults when compared to young adults (p<0.05). Furthermore, power of sway signal was substantially lower for both amputees and older adults. When compared to that of older adults, postural steadiness of amputees was more affected by the eyes closed condition, whereas older adults’ was more affected when sensory and proprioceptive information was perturbed by standing on foam.

**CONCLUSION:**

Our findings showed that fall risk is greater in amputees than in young adults without amputation. Additionally, amputees may rely more heavily on visual information than proprioceptive information for balance, in contrast to older and young adults without amputation.

## INTRODUCTION

Approximately 185,000 amputations occur in the United States per year, and about 2 million Americans currently live with a limb loss.^1–3^ The incidence of amputations per year ranges from 1.2 to 4.4 per 10,000 with a majority of amputations involving the lower limb.^4^ Falling is dangerous and debilitating, yet common, for individuals with lower extremity amputations.^5^ Nearly 50% of amputees experience an accidental fall within a year of their operation; over 40% of those falls result in serious injury, and over 19% require additional medical attention.^6^ As the amputee population ages, accidental falls become a greater problem. Increased rate of fall, reduced balance confidence, and increased fear of falling are reported following lower extremity amputation.^7,8^ Additionally, people with higher levels of amputation experience a higher rate of incidental falls.^7,9^

Postural steadiness, as measured by quantification of postural sway during quiet standing on a force platform, has been used frequently as a method of assessment of static balance and postural control. Postural steadiness may be an indicator of the quality of balance and postural control.^10–13^ Literature has linked time series^14,15^ and frequency domain^16–19^ variables of postural sway to balance. Additionally, it is reported that state of anxiety and fear of falling impact postural sway.^20,21^ Anxiety and fear of falling are psychological conditions that can lead individuals to avoid participation in activities.^7^ Activity avoidance due to the fear of falling can lead to reduced quality of life related to reduced strength, endurance, and balance, and can increase the risk for further health problems, including falls, in patients with lower extremity amputations.^22^

While most studies of postural steadiness focus on quantification of time series variables extracted from postural sway signals,^23^ others have suggested and utilized frequency domain variables of sway in parallel with time series variables to reveal more valuable information.^11,12,19^ Analysis of frequency content of a signal reveals underlying changes that often are not observed in time series. For instance, in the absence of movement, agonist and antagonist muscles may still be actively working against each other. Additionally, in the inverted pendulum model, that was introduced by Maurer and Peterka,^24^ ankle stiffness and noise may be increased simultaneously. In these cases, time series variables, e.g. velocity and displacement, do not show any changes. Power spectral density however, would provide information of the underlying conditions. As a result, it is often suggested that both time and frequency domain variables should be evaluated in assessment of postural steadiness. The purpose of this study was to determine change in both time and frequency domain variables of postural sway among lower extremity amputees as compared to healthy young and older adult controls.

## METHODOLOGY

Following approval of the Institutional Review Board (Northern Illinois University), a study was conducted to determine impact of lower extremity amputation on time series and frequency domain variables of postural sway. This study included 6 individuals with lower extremity amputation (2 unilateral trans-tibial [UTT], 1 bilateral transtibial [BTT], 2 unilateral trans-femoral [UTF] and 1 unilateral hip disarticulation [UHD]) with the average age of 51 (SD=16) years), 10 healthy young adults (age 25 (SD=1.6) years), and 10 healthy older adults (age 71.7 (SD=5.4) years). Amputee participants were included if they met the following criteria: a) were lower extremity amputees with more than one year of experience using a prosthetic limb, b) had a comfortable prosthetic limb about which they had no complaints, c) apart from lower extremity amputation, had no physical or mental disability that could potentially affect their balance, d) could ambulate without any assistance or use of an assistive device, and e) could stand upright independently for at least 10 minutes. Individuals with any visual deficits (apart from requiring corrective lenses) or vestibular deficits and those with a history of injury or surgery to the lower extremities within the past 6 months were excluded from the study. Healthy young and older adults were recruited if they were able to stand upright independently for at least 10 minutes and ambulate without assistance. Those with any physical or mental condition that could potentially impact postural control were excluded. Participants were asked to sign a consent form prior to participation in the study.

A Kistler force platform (Kistler Co., Winterthur, Switzerland) was used to collect position data of the center of pressure (COP) at 100 Hz. A LabVIEW program (National Instrument, Austin, Texas) was developed to collect postural sway data. Participants were randomly assigned to three standing conditions: a) eyes open, b) eyes closed and c) standing on Airex 2.5” thick foam balance pad (Airex Corporation, Somersworth, NH). The conditions of eyes closed and standing of foam were included to estimate changes in postural steadiness when visual and sensory information are diminished. Considering that vestibular, visual, and sensory information are typically relied upon to maintain upright posture, deterioration of any of these sources of information may reveal information regarding our dependency on the lost source. Each test condition was repeated three times. Test orders were block randomized, with each condition presented once in each block. During the study, participants were instructed to stand straight and static with arms on their sides (bare feet, heels together, 5-7 degrees of toe-out) on the force platform. Data was collected for 35 seconds (Fs=100). For the eyes-closed condition, researchers asked each participant to close his or her eyes and confirmed that eyes remained closed throughout the trial. While there were not any specific resting periods implemented between trials, participants were informed prior to the testing that they were welcome to request a rest time if they needed to. Additionally, during the trials participants were repeatedly asked if they wanted to rest. Several participants asked for the rest during the tests.

Anteroposterior and mediolateral time series data were filtered through a fourth-order zero phase Butterworth low-pass filter with cutoff frequency of 5 Hz. The first 8 seconds and last 2 seconds of data were cut off to remove any potential lead-in/lead-out effect. MATLAB and Toolbox Release 2012b (MathWorks, Inc., Natick, Massachusetts) were used to filter postural sway data and to compute variables of interest. SAS statistical software was used to conduct statistical analysis and to compare means between healthy adults and amputees. Time and frequency domain variables of postural sway were computed. Detailed explanation of computation methods for variables and equations are available in literature.^11,12,25–28^ Mean sway distance which represents the average sway from the mean position of the center of pressure was calculated for N data points as follows:


$$\text{Mean sway distance}=\frac{1}{\;\text{N}}\sum \sqrt{\text{AP}{\left[\text{n}\right]}^{2}+\text{ML}{\left[\text{n}\right]}^{2}}$$

Similarly, total excursion of sway as the total distance COP travels was computed by summation of distance between two consecutive data points:

Total excursion of sway


$$=\sum_{\text{n}=1}^{\text{N}-1}\sqrt{{\left(\text{AP}[\text{n}+1]-\text{AP}[\text{n}]\right)}^{2}+{\left(\text{ML}[\text{n}+1]-\text{ML}[\text{n}]\right)}^{2}}$$

Velocity of the sway was calculated by dividing total excursion over the time:


$$V\text{elocity of the sway}=\frac{\text{ total excursion of sway }}{\text{ total time }}$$

Power of sway signal was computed as the integrated area of power spectrum, and 95% power frequency was determined as the point below which 95% of the total power is placed.^12^

A linear mixed model with the random effects for subjects and subjects × condition was used to compare the means. The least square means for the three groups of amputees/older adults/young adults × condition and their pairwise differences were computed for this model.

## RESULTS

**Figure 1:**
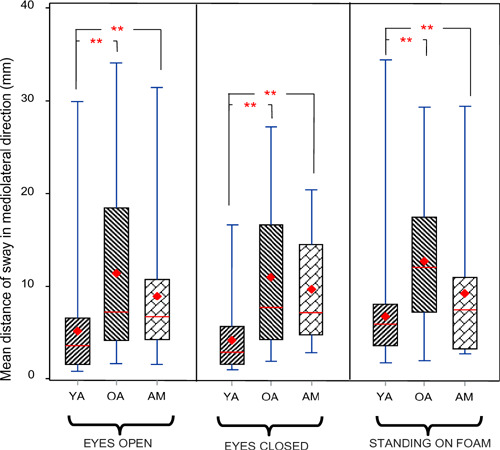
Comparison of mean distance from the mean center of pressure in mediolateral direction during static standing for three different conditions of eyes open, eyes closed, and standing on foam. Diamond shape and solid line indicate mean and median of the data respectively. Data presented for three groups of amputees (AM), older adults (OA) and young adults (YA). Asterisks (**) denote statistically significant differences (p<0.05). Our secondary analysis for the resultant power of sway signal showed a similar pattern as our original analysis. Resultant power of sway was significantly different between the three groups (amputees, young adults, and older adults) (p=0.009) and between the three conditions of standing (p=0.01). Older adults demonstrated a higher level of power when compared to young adults. This difference was not statistically significant for the eyes open condition and eyes closed conditions, but significantly higher for older adults when standing on foam (p=0.002). Power of sway signal for the older amputee group was higher than for young adults and lower than for older adults under conditions of eyes open, eyes closed and standing on foam. Analysis of the resultant mediolateral 95% power frequency showed that this value is significantly lower for both older amputees and older adults without amputation when compared to young adults (p<0.05) for eyes-open and eyes-closed conditions. This difference however, was not statistically significant when participants were standing on foam.

Comparison of older adults, younger adults, and amputees on the three conditions of standing eyes open, eyes closed, and on foam revealed significant differences between groups. Mean mediolateral (ML) sway distance from the COP was significantly increased by both amputation and aging (P<0.0001). Tukey-Kramer post hoc analysis revealed that amputees’ COP deviated a significantly higher distance from the central point than did COP of young participants (Figure 1), particularly with eyes closed (p=0.02). Similarly, older adults swayed a greater distance mediolaterally than did young adults (p=0.001). The difference between older adults and amputees however, was not statistically significant, even though this value was higher for older adults. Furthermore, the difference in total excursions of sway during static standing was also significantly different between the three groups (amputees, young adults, and older adults) (p<0.0001).

**Figure 2:**
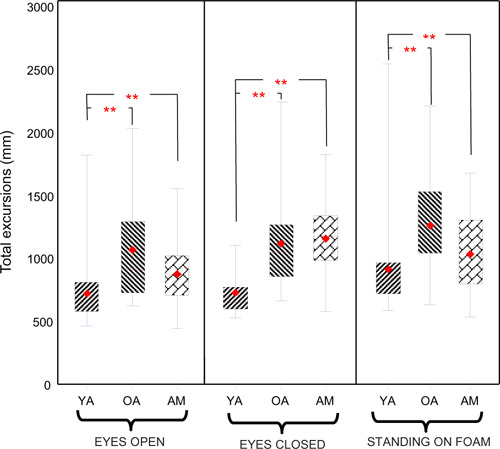
Comparison of total excursion of sway for three different conditions of eyes open, eyes closed, and standing on foam. Diamond shape and solid line indicate mean and median of the data respectively. Data presented for three groups of amputees (AM), older adults (OA) and young adults (YA). Asterisks (**) denote statistically significant differences (p<0.05).

**Figure 3:**
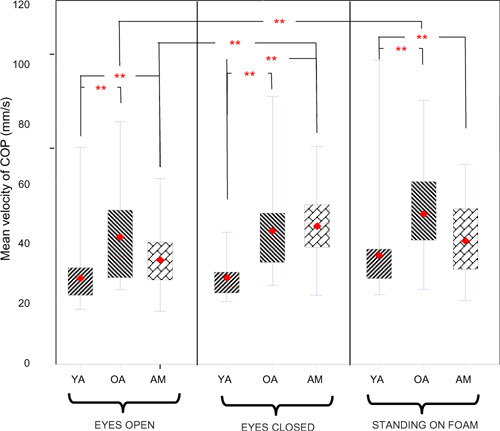
Figure depicts mean velocity of sway for three different conditions of eyes open, eyes closed, and standing on foam. Diamond shape and solid line indicate mean and median of the data respectively. Data presented for three groups of amputees (AM), older adults (OA) and young adults (YA). Asterisks (**) denote statistically significant differences (p<0.05). Note variation of mean velocity when amputees and older adults are compared for the conditions of eyes closed versus standing on foam.

Post hoc analysis showed greater total excursions among older adults when compared to young adults (p<0.05) (Figure 2). When older adults were compared to amputee participants, however, the value of excursions was significantly higher for amputees (p=0.0008) only when participants were standing eyes closed. Mean velocity of sway was different between groups (p<0.0001). Older adults showed higher velocity of sway when compared to young adults in all conditions of standing, i.e., eyes open (p=0.002), eyes closed (p=0.0005), and standing on foam (p=0.001). While amputees swayed at higher velocity than did young adults, their velocity of sway was still lower than older adults’ in eyes open and standing on foam conditions and slightly higher in eyes closed condition. These differences, however, were not statistically significant. When eyes were closed, amputees showed a substantially higher velocity of sway than did young adults (p=0.0008) (Figure 3).

Velocity of sway also varied based on condition of standing for all groups (p=0.0176). Amputees’ sway velocity was greatest with eyes closed, followed by standing on foam, and least with eyes open. These differences in sway velocity for the amputee group were statistically significant only for eyes closed vs. eyes open (p=0.024). This pattern however, was different for older adults. Older adults showed their highest velocity of sway when standing on foam. There was no statistically significant difference in sway velocity for older adults with eyes closed vs. eyes open, or eyes closed vs. standing on foam. The difference in velocity of sway was significant, however, when foam standing was compared to eyes open (p=0.0446).

**Figure 4:**
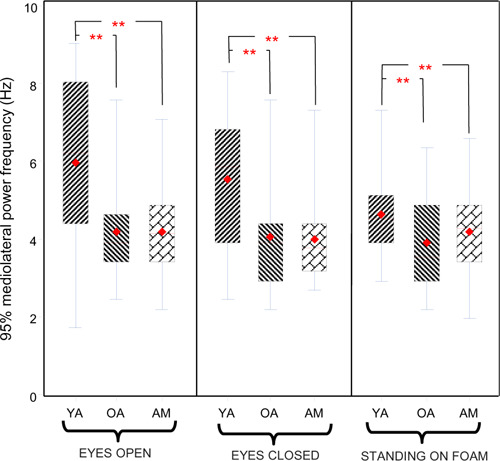
Comparison of 95% mediolateral power frequency of sway signal for three different conditions of eyes open, eyes closed, and standing on foam. Diamond shape and solid line indicate mean and median of the data respectively. Data presented for three groups of amputees (AM), older adults (OA) and young adults (YA). Asterisks (**) denote statistically significant differences (p<0.05).

Resultant power of sway signal was also significantly different between the three groups (amputees, young adults, and older adults) (p=0.0074) and between the three conditions of standing (p=0.025). No significant interaction between groups and conditions of standing was noted. When young and older adults were compared, Tukey-Kramer post hoc analysis showed that older adults demonstrated a higher level of power. Although this difference was not significant for the eyes open condition, it was nearly significant for the eyes closed (p=0.05) and significant for standing on foam (p=0.0014) conditions. Power of sway signal for the amputee group was higher than for young adults and lower than for older adults under conditions of eyes open, eyes closed and standing on foam. This difference however, was not statistically significant. Further analysis of our data showed that the resultant mediolateral 95% power frequency was significantly lower for both amputees and older adults when compared to young adults (p<0.05) for eyes open and eyes closed conditions (Figure 4). Lower power of amputee and older adults however, was not significant compared to young adults for standing on foam condition.

Since our amputee population consisted of both young and older adults, we performed a secondary analysis on our data, eliminating data for amputees under the age of 60. We anticipated that this change would lead to a more homogenous sample of amputees who were all older adults. Therefore, comparison of amputees with older adults and young adults may be more telling. As a result, the amputee group in our secondary analysis consisted of 3 amputees (age 62 (SD=3.8) years). Similar to our previous findings, we noticed that older amputees sway at a significantly higher level with eyes closed than do young adults (p=0.046), but the sway distance, although higher, does not differ substantially when compared to older adults without amputation (p>0.05). Furthermore, we noticed that even though older amputees performed significantly higher total excursion of sway when compared to young adults (p=0.001), their total excursion was not substantially different from older adults in closed-eyes conditions. This latter finding was not in agreement with our original findings. Pattern of changes in sway velocity was similar to our original analysis. We noticed that older adults showed higher velocity of sway when compared to young adults in all conditions of standing, i.e., eyes open (p=0.002), eyes closed (p=0.0006), and standing on foam (p=0.002). When eyes were closed, older amputees showed a substantially higher velocity of sway than did young adults (p=0.0013). The difference of sway velocity among older adults and amputee was not statistically significant.

We performed an additional analysis on our data by removing the two individuals with hip disarticulation and bilateral transtibial amputation and included only those with unilateral transfemoral and transtibial amputation for analysis.

Considering that static balance is mainly controlled through ankle and hip strategies,^29^ we attempted to create a more homogenized sample by removing two participants from the sample. We anticipated, unlike other participants, bilateral amputees may not have a chance to compensate for the loss of ankle strategy through the sound limb. Although inability to perform hip strategy in individuals with hip disarticulation is yet to be determined, we also excluded this participant to avoid any potential bias.

**Figure 5:**
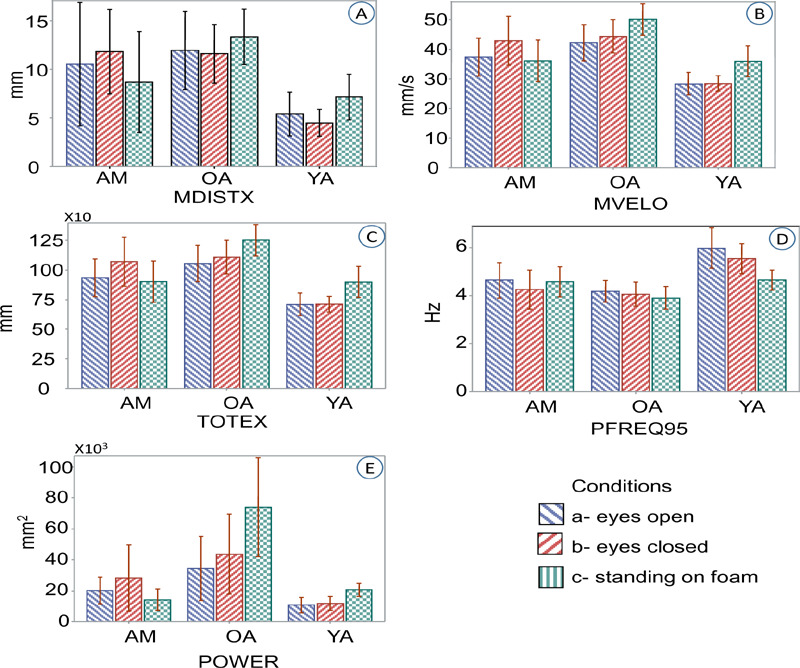
Graph depicts mean values and 95% confidence limits for amputee group (AM), older adults (OA) and young adults (YA). Data represents amputee participants with unilateral transtibial and transfemoral amputation (n=4). **A**: Variables presented are mean distance of sway in mediolateral direction (MDISTX); **B**: mean velocity of COP (MVELO); **C**: Total excursions (TOTEX), **D**: 95% mediolateral power frequency (PFREQ95); **E**: Resultant power of sway (POWER).

Comparison of means and confidence limits for time and frequency domain variables showed almost a similar pattern as previous analysis. When older adults and young adults were compared with amputees, except for resultant power, amputees’ variables were closer to those of older adults than young adults (Figure 5). The mean of resultant power, however, was closer to the mean power of young adults than older adults (Figure 5E).

Further analysis showed that participants with amputation swayed at a significantly higher velocity when compared with young adults (p=0.012) during eyes closed condition. On the other hand, amputees swayed at a significantly lower velocity when compared to older adults (p=0.015) during standing on the foam condition. Similarly, amputees total excursion of sway was higher than young adults in eyes closed trials (p=0.012) and less than older adults in trials of standing on foam (p=0.015). When power of sway signal during standing on foam condition were compared, amputee participant generated significantly less power during static standing comparing with older adults (p=0.002).

**Table 1:** Table consists of demographic information on study participants. Participants are presented as non-amputees (NAM), right/left trans tibial (RTT/LTT), bilateral trans tibial (BTT), left Trans-femoral (LTF) and left hip disarticulation(LHD). Most study participants with amputation was using Dynamic Response (DR) prosthetic feet.

Participant	Gender	Age	Height (cm)	Weight (Kg)	Condition	Res. Limb. Length	Reason	Prosthetic foot
1	M	26	172	67.1	NAM	--	--	--
2	M	29	171	60.3	NAM	--	--	--
3	M	24	181	80	NAM	--	--	--
4	M	24	188	76.1	NAM	--	--	--
5	F	25	160	65.2	NAM	--	--	--
6	F	25	153	58.4	NAM	--	--	--
7	M	77	172.5	66	NAM	--	--	--
8	F	72	166.6	68.8	NAM	--	--	--
9	M	25	184.5	91.1	NAM	--	--	--
10	M	24	187	75.3	NAM	--	--	--
11	F	24	167.5	69.5	NAM	--	--	--
12	F	24	159	68.3	NAM	--	--	--
13	M	69	172	79.5	NAM	--	--	--
14	F	68	167	62.9	NAM	--	--	--
15	M	70	166	78.7	NAM	--	--	--
16	M	77	174.5	86.9	NAM	--	--	--
17	F	82	160.5	67.1	NAM	--	--	--
18	M	65	174.8	104.4	NAM	--	--	--
19	M	71	177.5	94.1	NAM	--	--	--
20	F	66	157.6	82.1	NAM	--	--	--
21	M	30	196	100.5	RTT	10	Trauma	DR
22	M	58	173.4	101	LTT	16.5	Trauma	DR
23	M	30	185.5	84.4	LTF	36	Trauma	DR
24	M	61	177	100.1	LTF	37	Disease	DR
25	F	60	160.51	84.9	LHD	0	Disease	DR
26	M	67	182.5	92.1	BTT	20R/25L	Disease	SACH

## DISCUSSION

It has been documented that mediolateral stability significantly correlates with the risk of falling in older adults.^30^ Winter et al. previously highlighted the importance of mediolateral sway during quiet standing.^31,32^ It has also been shown that mediolateral sway is increased in fallers when compared to non-fallers.^33^ Our results suggest that lower limb amputation significantly affects postural steadiness. Comparison of our finding with young and older adults also revealed important aspects of postural control in lower extremity amputees. To our knowledge, no other studies have compared postural stability of lower extremity amputees against that of young and older adults. Our results showed that while older adults swayed mediolaterally more than did amputees, the difference was not statistically significant. Both amputees and older adults, however, swayed significantly more than did young adults.

This variation, coupled with others’ findings relating sway to falls, supports the conclusion that both older adults and lower limb amputees are prone to falling. Our study finding is in agreement to those of Buckley et al,^34^ who reported increased sway distance in a group of 6 trans-tibial/transfemoral amputees.

Similarly, total excursions of the center of pressure was higher for both older adults and amputees. When standing was challenged by a compliant (foam) surface, we noticed greater total excursions of sway in older adults, when compared to other conditions. Further analysis of sway velocity augmented this finding.

Velocity of sway is recognized as one of the most important variables of sway analysis that can determine effects of aging on balance.^12^ Our results showed that older adults and amputees sway at a significantly higher velocity than do young adults (p<0.0001). Our post-hoc analysis did not reveal any significant difference in sway velocity between amputees and older adults. We also noted that with both amputees and older adults, the sway velocity increased when the condition was changed from eyes open to either eyes closed or standing on foam. It is particularly interesting, however, that the pattern of velocity is different between amputees and older adults when evaluating the three standing conditions. The two conditions of eyes closed and standing on foam are primarily designed to diminish visual and somatosensory/proprioceptive information, respectively, to the postural control system. It has been well documented and also seen in our own data that loss of any one of these sources of information for postural control leads to an increased sway and sway velocity in static standing.^35^ In our study, when amputees and older adults were compared, amputees swayed most when their eyes were closed, whereas older adults swayed more when they were standing on foam. Amputees’ sway velocity with eyes closed was significantly more than with eyes open (p=0.024); whereas, sway velocity did not increase significantly from eyes open to foam standing (p=0.19). On the other hand, for older adults, increase of sway velocity was significant when eyes open was compared to foam standing (p=0.044), while the increase in sway velocity was not significant when eyes open was compared to eyes closed (p=0.59). Both groups had corrected visual acuity of 20/20 with no known sensory deficits or physical or mental conditions that could potentially affect their balance. This may indicate that amputees are more dependent on visual information, whereas older adults are more dependent on somatosensory and proprioceptive information, to control their balance. The increased dependency of lower extremity amputees on visual input is also reported by Arifin et al.^36,37^ The results of this study suggest that lower limb amputation significantly affects postural steadiness. Additionally, it was noted that mediolateral postural sway and velocity of sway of lower limb amputee participants of this study were slightly less than those values for older adults. Furthermore, while both amputees and older adults represent a diminished postural steadiness, older adults’ steadiness is challenged more when standing on the foam, while amputees’ steadiness is more challenged when standing with eyes closed. It appears that when older adults and amputees are compared, most likely older adults are more dependent on their sensory information, while amputees are more dependent on their visual information. in a sample of trans-tibial amputees, although no comparisons were made between amputees and young or older adults in this study.

## CONCLUSION

Also compared were the resultant mediolateral 95% power frequency among the study participants. The 95% power frequency is an estimate of the extent of the spectral content and indicates the frequency below which 95% of the integrated area of power spectrum resides. More detailed definition and method of calculation is explained elsewhere.^12,38^ Power spectral density is known to indicate the underlying mechanism of postural control.^12^ Therefore, the study suggests that the underlying mechanism of postural control for both amputees and older adults changes when eyes were closed, but not when they were standing on foam.

### Limitations

There are several limitations acknowledged with respect to the generalizability of the study results. First, the study involved a small number of amputee participants: 6 individuals with lower extremity amputation and 20 healthy controls. Although the findings suggest that amputees have an increased risk for falls and that they may rely heavily on visual input for postural control, replication should be sought with a larger sample. It is to be noted that the original sample included both young and older adults. In the secondary analysis, however, the data of amputees older than 60 years was only included. Although this change made the study sample more homogenous, it reduced the sample size even more. Additionally, all amputee data was combined, regardless of the level of amputation or, in the case of one participant, bilateral vs. unilateral amputation. As a result, this combination may have affected the results, but the level of this impact is yet to be investigated. Considering that in the inverted pendulum model of postural control, as suggested by Maurer and Peterka,^24^ postural sway is substantially controlled at the ankles. In fact, in a study of 8 unilateral trans-femoral amputees, Hlavackova et al.^39^ showed that the sound limb is most responsible for sway velocity when compared with the amputated side. In the current study, participants had different levels of amputation with the common characteristics that they were all missing their ankle. Nevertheless, further studies with a larger and more homogenous sample would be warranted to compare postural sway data between individuals with lower extremity amputation at different levels.

## DECLARATION OF CONFLICTING INTERESTS

Author do not have any conflict of interest to disclose.

## SOURCES OF SUPPORT

N/A

## ETHICAL APPROVAL

This study approved (HS11-0407) by the Institutional Review Board, Northern Illinois University. Participants were asked to sign a consent form prior to participation in the study.
